# Research on Cement Slurry Using Silica Fume Instead of Fly Ash

**DOI:** 10.3390/ma15165626

**Published:** 2022-08-16

**Authors:** Yue Wu, Wei-Guo Qiao, Yan-Zhi Li, Hui-Ni Liu, Chao Tang, Shuai Zhang, Xiao-Li Zhang, Ji-Gang Lu, Peng-Cheng Chen

**Affiliations:** 1 Shandong Provincial Key Laboratory of Civil Engineering Disaster Prevention and Mitigation, Shandong University of Science and Technology, Qingdao 266590, China; 2 School of Civil Engineering, Ludong University, Yantai 264025, China

**Keywords:** silica fume, cement slurry, properties, waste recycling, feasibility

## Abstract

Ordinary cement is not environmentally friendly, has high cost and lacks superior performance. Many scholars use various admixtures to adjust the properties of cement slurry, but admixtures are usually not environmentally friendly, and it is difficult to ensure that the properties after deployment meet engineering requirements. In this study, a variety of admixtures were obtained using the environmental protection method, and the optimal mixing ratio was analyzed by combining the entropy weight method and the Taguchi grey relational analysis method. The developed cement slurry was compared with conventional slurry from both macroscopic and microscopic aspects. Aiming at the problem that previous scholars lacked the engineering feasibility verification of the developed slurry, this study combined the constitutive equation regression analysis method, discrete element numerical simulation and other methods to study various actual engineering conditions. The results show that the optimal mix ratio of silica fume cement slurry has good permeability characteristics under the conditions of different roughness, grouting pressure and confining pressure. At the same time, under different geological temperatures and different erosive liquid states, the cement slurry stone body shows good properties of reinforcement materials.

## 1. Introduction

Grouting, as an important means of strengthening water blocking and disaster control in geotechnical engineering, can effectively improve the structure and mechanical properties of complex strata such as fractured rock mass and fractured zone, and is widely used in tunnel engineering, mining engineering, dam engineering and other underground projects, where various water hazards or unstable rock mass reinforcement are treated [1,2,3]. Among them, the grouting material, as the key to determine the success or failure of grouting technology and an important factor affecting economic indicators [4,5], has received extensive attention from scholars at home and abroad [6,7].

Ordinary Portland cement is widely used as the main bonding material for the preparation of concrete, mortar, grouting materials, etc. However, Portland cement manufacturing has become one of the major contributors to CO_2_ emissions over the past few decades [8,9]. In the cement industry, the use of supplementary cementitious materials (SCMs) such as fly ash and slag as a partial replacement for cement has become a well-known approach. It not only reduces the consumption of natural resources and carbon dioxide emissions during the manufacture of cement, but also reduces the waste of industrial by-product resources. However, cement-based materials containing a large number of SCMs will lead to a slow early hydration reaction and affect their early performance [10,11,12,13]. For example, fly ash cement slurry has been widely used in water plugging reinforcement and anti-seepage projects under different hydrogeological conditions due to its low price, good impermeability, good fluidity and non-toxicity. However, the stone body strength of the slurry with fly ash is reduced, the stability is poor, and the erosion resistance is insufficient, and it is difficult to meet the demands of grouting reinforcement projects that require high erosion resistance and stone body strength. To meet the requirements for the performance of these materials containing SCMs, researchers have obtained many valuable breakthroughs and progress.

In recent years, many scholars have carried out valuable research on the application of nanomaterials in cement-based materials [14,15,16,17], providing a new idea and new method for improving the performance of cement-based materials containing SCMs [18,19,20]. Compared with other nanomaterials, nano-CaCO_3_ has the characteristics of high efficiency and low price, and there are many research results for nano-CaCO_3_ modified cement-based materials. Sato and Beaudoin [21] pointed out that the addition of nano-calcium carbonate significantly accelerated the early hydration rate of FIPC (fine-grained Portland cement), mainly due to the seeding effect and C-S-H nucleation of nano-calcium carbonate particles, which led to the enhancement of strength. Li et al. [22] have shown that the density of concrete is significantly improved after adding nano-calcium carbonate, but if the addition amount is too large, it will lead to local relaxation and expansion of concrete. Qian et al. [23] showed that nano-CaCO_3_ improves the compressive strength of cement-based materials mainly by refining the crystal form of hydration products and reducing porosity through its crystal nucleation, thereby improving the microstructure.

Moreover, previous studies did not focus on environmental protection and did not verify the feasibility of the project. In terms of mineral admixture selection, studies by scholars have shown that the production source of silica fume is more environmentally friendly (compared to the production of ash) and the cost is low [24]. Silica fume has the same chemical characteristics as fly ash: a spherical material that promotes rolling between particles. At the same time, the mechanical properties of silica fume are better than those of fly ash [25,26,27]. That is to say, compared with fly ash, silica fume is optimized in terms of environment, economy, mechanics and other objectives. Therefore, this study selects silica fume to replace part of the fly ash.

This study effectively alleviates the problems of high cost and environmental protection of traditional cement slurry. A variety of technical means are used in combination, and the feasibility of the developed slurry is fully verified by examining the macro and micro perspectives, laboratory and practical applications, numerical simulation and theoretical derivation. Based on the theory of synergy and complementarity, a variety of admixtures for improving cement slurry were obtained from waste on the basis of consulting a large amount of research and development literature on cement-based materials. Using theoretical analysis, laboratory test methods, combined with advanced methods such as SEM (Scanning Electron Microscope) and XRD (X-Ray Diffraction), the research and development of cement-based composite grouting materials was carried out. The basic properties of the slurry and the mechanism of cement hydration were studied. The proportion of cement slurry was optimized by designing an improved Taguchi orthogonal test, and the significant relationship between the content change of the admixture and the performance of grouting material was determined. An environmentally friendly cement slurry with excellent performance was obtained. Through experiments and numerical simulations, the multiple properties of the cement slurry under different engineering conditions were analyzed. Research results indicate that the cement slurry has good reliability and feasibility and can be applied to a wide range of construction projects.

## 2. Materials and Methods

Materials adopted in the research: FIPC, silica fume (SF), fine particle size fly ash (FIPA), mCaCO_3_·nH_2_O (colloid, abbreviation for CH) and polycarboxylate superplasticizer (PS).

### 2.1. Materials

#### 2.1.1. FIPC

**Figure 1:**
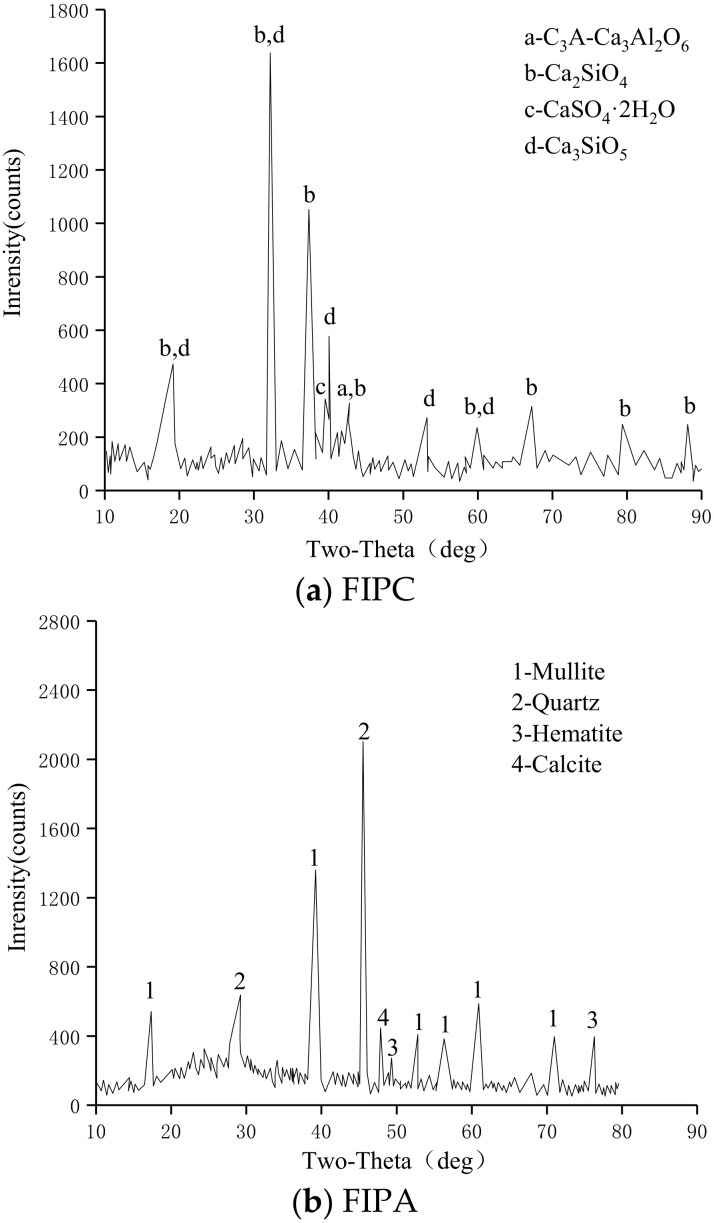

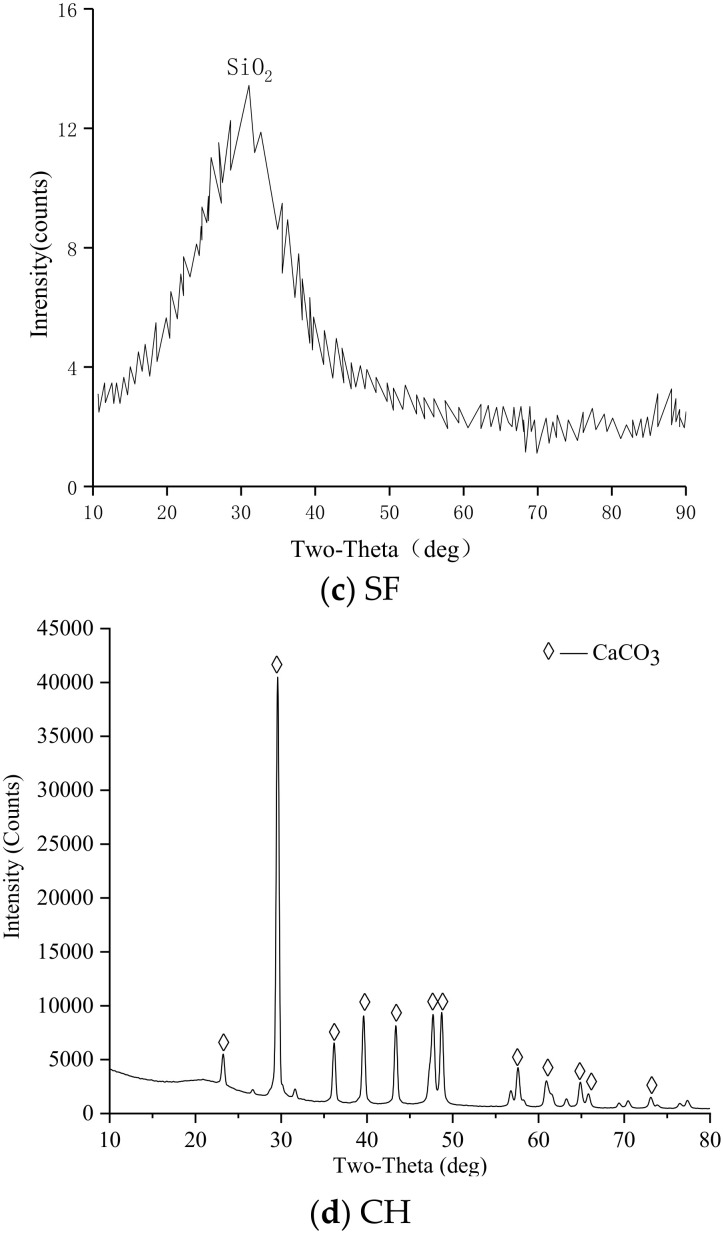
XRD pattern of FIPC, FIPA, SF, CH.

To make the cement react sufficiently in the research process and be able to adapt to the smaller cracks in the rock, the P.O42.5# cement was ground into a smaller micron level (2.39~9.51 μm) by a grinder to obtain FIPC. Its main chemical composition and basic physical properties are shown in Table 1. The XRD pattern of FIPC is shown in Figure 1a.

#### 2.1.2. FIPA

Combustion waste was obtained from factory waste and ground to micron level (1.02–8.62 μm) to obtain FIPA, which has the properties of aluminosilicate glass [28]. The chemical composition of FIPA is shown in Table 1. The XRD pattern of FIPA is shown in Figure 1b.

#### 2.1.3. SF

Silica fume with large particle size is obtained from the waste of the silicon refining plant and ground to the micron level (2.18~10.24 μm) to obtain SF. Its chemical composition is shown in Table 1. The XRD pattern of SF is shown in Figure 1c.

#### 2.1.4. CH

CH is obtained by:

1. Obtaining CaCO_3_ by using chemical methods from limerock, etc.;
2. The acquired CaCO_3_ is processed into nano-scale powder by a grinder;
3. Obtaining mCaCO_3_·nH_2_O by sol-gel method, hereafter referred to as CH; mCaCO_3_·nH_2_O is a nanoscale sol.

The use of waste limestone to prepare CH would not only improve the problem of wasteful economic costs in processing large amounts of waste limestone, but also play a positive role in the early hydration and consolidation strength of the slurry.

The technical indicators of CH are shown in Table 2. Figure 1d shows the XRD diffraction pattern of CH, which shows that its crystal phase material is mainly calcium carbonate.

#### 2.1.5. PS

**Figure 2:**
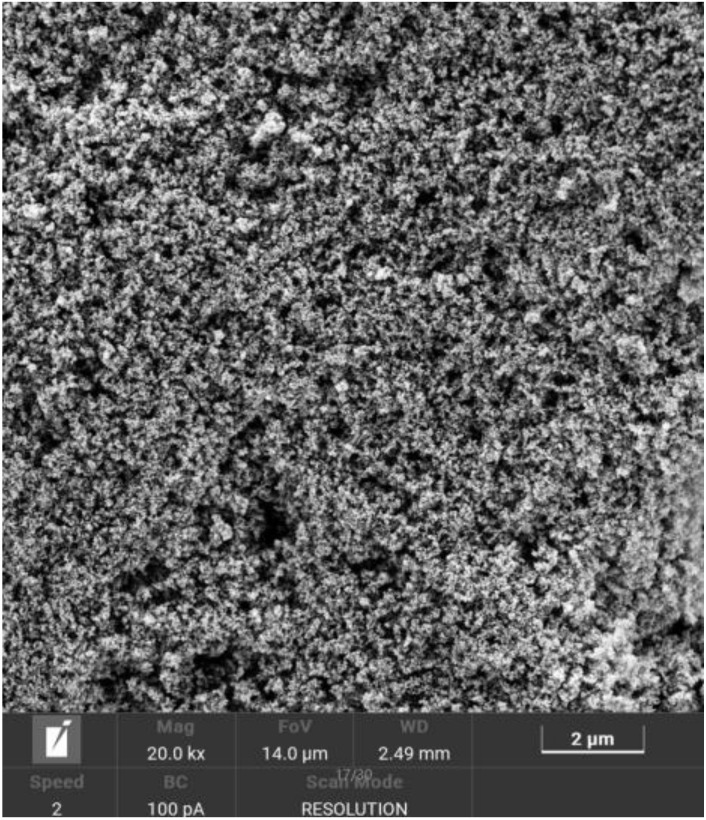
SEM image of PS.

The PS used in this study is produced by a chemical company in Shanghai. The appearance is white powder, the specific gravity is 1.08, the active ingredient is ≥90% and it has the effect of air entrainment and retardation. A certain number of air bubbles can enhance the fluidity of the cement slurry, but too many air bubbles will adversely affect the mechanical properties of slurry consolidation. Therefore, the air-entraining effect of the water-reducing agent should also be considered when applying the water-reducing agent. Figure 2 is a SEM image of PS. In Figure 2, the PS contains a large number of carboxylic acid groups to improve the performance of cement slurry.

### 2.2. Methods

The main properties of cement-based slurry studied in this paper are: viscosity (VSI), bleed capacity (BC), fluidity (FL), setting time (ST), compressive strength (CS) and flexural strength (FS) of stone bodies at different ages (3 d, 28 d). The specific test methods for various properties of grouting materials are as follows:

Flow characteristics: the VSI of the slurry was measured using an SNB-2 digital rotational viscometer. FL was tested using a truncated cone die and a glass plate.

Congealing properties: BC was tested using the traditional graduated cylinder method. The ST test uses a Vicat instrument. In this study, only the initial setting time was used as an example to illustrate the coagulation characteristics.

Mechanical properties (FS and CS): Tested by using a flexural testing machine and a compression testing machine, respectively.

Microscopic testing (XRD and SEM): Routine experiments were performed using XRD and SEM equipment.

### 2.3. Experimental Scheme

The experimental test factors are:

A: Water-solid ratio (WSR).
B: FIPA content.
C: SF content.
D: PS content.
E: CH content.

The experimental factor level table is shown in Table 3.

According to the idea of the Taguchi-grey relational analysis method in Wu et al. [13], the five-factor and four-level orthogonal test was used to optimize the design of the cement-based composite slurry mix ratio. The Taguchi orthogonal test table is shown in Table 4.

## 3. Results

### 3.1. Orthogonal Test Results

The slurry performance test was carried out according to Table 4. The test results of various properties of the slurry are listed in Table 5. In this study, the signal-to-noise ratios (SNR) of fluidity and stone body strength were calculated by using the large-scale characteristics, and the corresponding SNR of the other properties were calculated using the characteristics of the small-scale characteristics (based on Wu et al.’s [20] research idea).

### 3.2. Flow Characteristics (VSI and FL)

**Figure 3:**
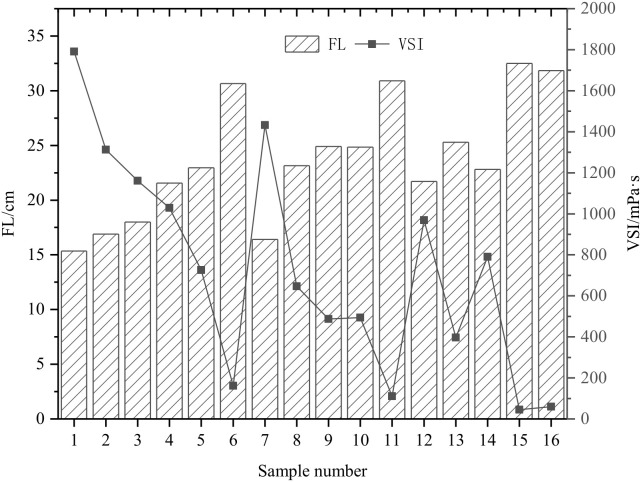
Slurry FL and VSI orthogonal test results (sample numbers 1–16 in the figure correspond to A_1_–D_4_, respectively, the same below).

Good flow characteristics (VSI and FL) ensure the grouting material’s castability and the best grouting effect. The orthogonal test results of slurry VSI and FL are shown in Figure 3.

On-site construction requires the grouting material to have good fluidity. When the fluidity of the slurry is too good, the slurry is lost in large quantities due to insufficient adhesion during the grouting process, resulting in unnecessary waste of resources; when the fluidity of the slurry is insufficient, in the process of flow and diffusion, a larger grouting pressure is required to resist the resistance of the slurry. The effect of time increases the viscosity of the slurry, which makes it difficult for the slurry to pass through small cracks and affects the grouting effect. In addition, the greater the fluidity of the slurry, the more difficult is the phenomenon of segregation and stratification occurs, and good fluidity can ensure the injectability and grouting effect of the grouting material.

The sample range (SR) analysis results for the SNRs of the FL and VSI are shown in Table 6. According to the range analysis of the SNR of the FL, the order of the influence of the five factors on the SNR of the FL is: A > D > C > E > B. A has the greatest impact on it, and B has the least impact on the SNR of the FL. According to the range analysis of the SNR of the VSI, the order of the influence of the five factors on the SNR of the VSI is: A > D > C > B > E.

**Figure 4:**
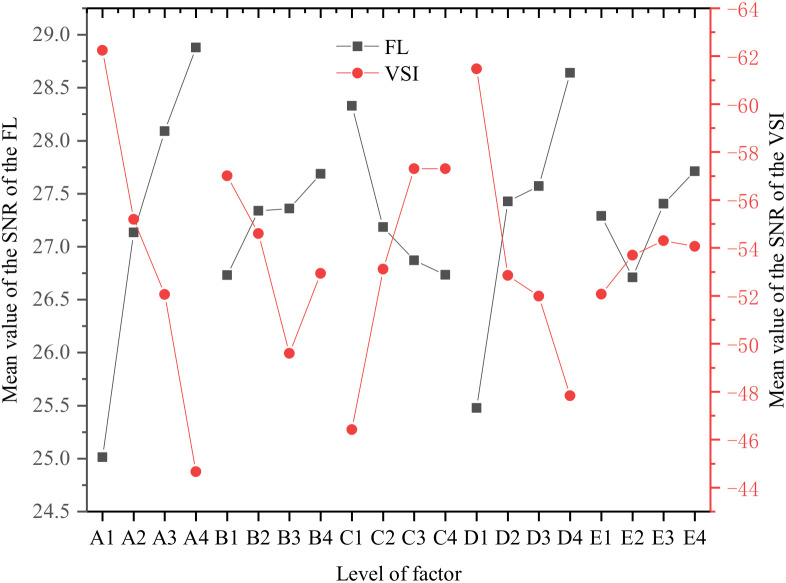
Mean value of the SNR of VSI and FL at different factor levels.

To more intuitively analyze the influence of the dosage levels of each factor on the FL and VSI of the slurry, the mean value of the SNR of the FL and VSI of the slurry under each influencing factor was drawn, as shown in Figure 4.

According to Figure 4, Factor A has the most significant impact on FL and VSI. As A increases, FL also increases and VSI decreases. The main reason is that the amount of water required for the hydration reaction of the slurry is certain, and the excess free water will always exist in the slurry, thereby significantly reducing the VSI of the slurry. With the increase of PS content, the slurry FL increased while the VSI decreased. The “comb-like structure” of PS enables it to be adsorbed on the surface of cement particles. In addition, the lubricating and dispersing effects of PS avoid the agglomeration of the cement slurry, which can effectively decrease the VSI of the cement slurry, thereby increasing the FL of the cement slurry.

The morphology of FIPA is spherical microbeads, its surface is smoother than cement, and its adsorption force to water molecules is small. These morphological characteristics reduce the water demand of the slurry, which leads to an increase in the excess free water content when Factor B increases, and the slurry FL increases accordingly. The larger Factor C is, the larger the VSI of the slurry is, which is due to the rapid dissolution of some small particles after SF is in contact with water. The SiO^2−^ rich and Ca^2+^ poor gels in the solution form an adhesion layer on the surface of SF particles. After a certain period of time, the gel adhesion layer begins to dissolve and reacts with Ca(OH)_2_ produced by cement hydration to form C-S-H gel, thereby increasing the VSI of the slurry. With the increase of Factor E, VSI showed a trend of increasing first and then decreasing. On the one hand, due to the filling effect of CH, the interstitial filling water of the cement clinker is replaced, which increases the free water molecules. On the other hand, CH has a large specific surface area and a large contact area with water molecules, which can adsorb a large number of water molecules, thereby increasing the VSI of the slurry, which is the same as the research conclusion of Qiu et al. [29].

According to the index of grouting material FL, the optimal ratio of each material component is: A4B4C1D4E4. That is, the grouting material has the maximum FL at this ratio. From the perspective of grouting material VSI, according to the principle that the VSI degree of grouting material be as small as possible within a reasonable range, the optimal ratio of each material component is: A4B3C1D4E1.

### 3.3. Consolidation Characteristics (ST)

**Figure 5:**
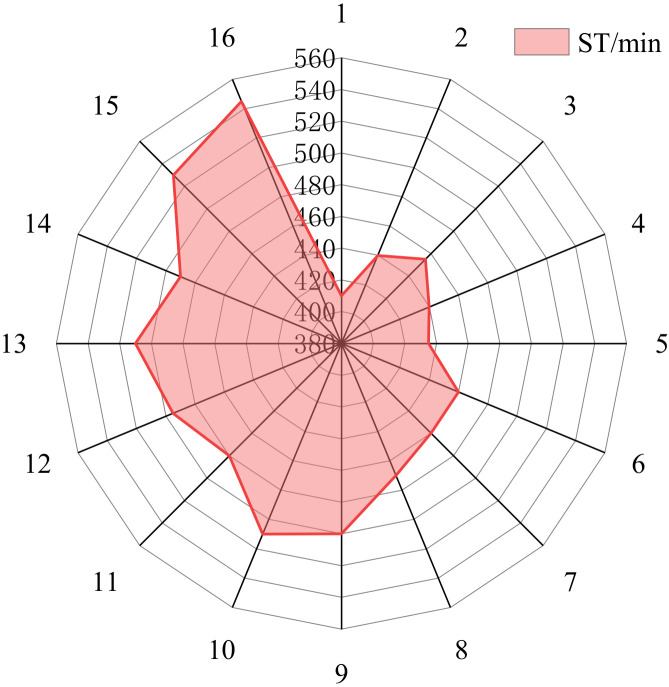
ST orthogonal test results.

The results of the ST orthogonal test are shown in Figure 5, and the SR of the SNR of ST analysis results are shown in Table 7.

Analysis of Figure 5 and Table 7 shows that the order of the influence of the five factors on ST is: A > B > E > C > D. Factor A has the greatest effect on ST and Factor D has the least significant effect on ST, which is consistent with the research of Zhang et al. [4].

**Figure 6:**
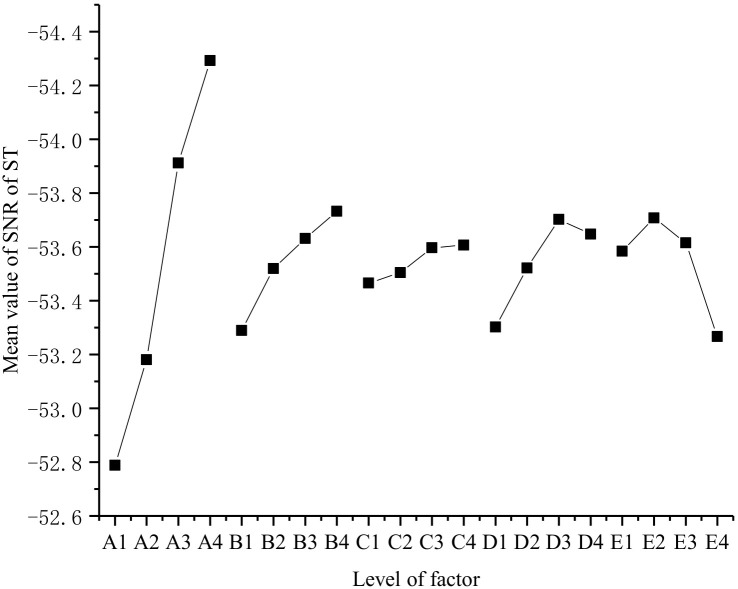
Mean value of the SNR of ST at different factor levels.

From Figure 6, factor A has the most significant influence on ST, followed by factor B. With the increase of Factor A and Factor B, ST showed a continuously extending trend. With the increase of CH content, ST showed an increase first and then decrease. When the content of CH is 1%, the SR of ST reaches the maximum value, which is the same as the research conclusion in the existing literature [30]. The reason for this is the filling and nucleation of CH. The particle size of CH is very small and it can fill the voids of cement and other particles to replace water, which reduces the hydration rate and prolongs ST. However, when the content of CH continued to increase, CH provided a large number of nucleation sites for hydration products, which accelerated the hydration reaction process.

For the ST, the optimal ratio of each material component is: A1B1C1D1E4, and the grouting material has a shorter ST under this ratio.

### 3.4. Slurry Stability (BC)

**Figure 7:**
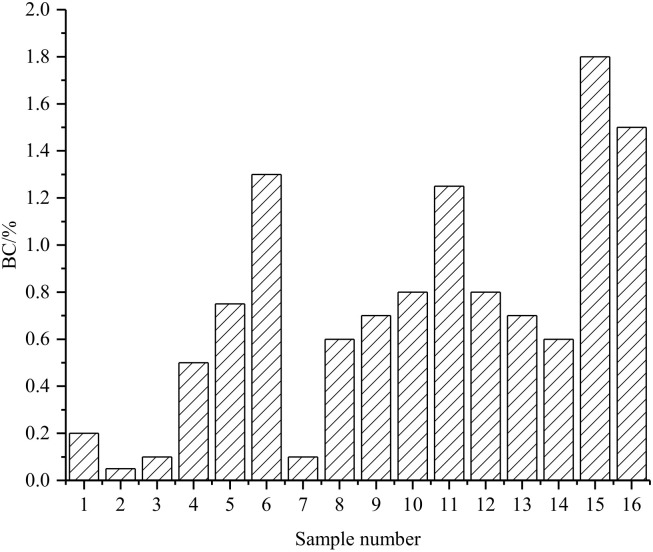
BC orthogonal test results.

The BC is an important indicator to characterize the stability of the grouting material. Cement-based grouting material is a solid–liquid two-phase fluid with complex fluid properties. The stability of the grout directly affects the integrity and strength of the broken rock mass after grouting. The stability of the grouting material is usually expressed by BC. The slurry with low BC has good mechanical properties after coagulation, and the lower the BC, the greater the stone rate. Generally, slurries with a BC of less than 5% are considered stable slurries. The results of the BC orthogonal test are shown in Figure 7. Analysis of Figure 7 shows that the BC of the slurries are all lower than 5% under each group ratio. This shows that the slurries under each composition ratio in this test scheme belong to stable slurries. Among them, the BC of the second group of proportioning slurry was the minimum of 0.05%, and the BC of the 15th group was the maximum of 1.8%. In this experiment, the BC of the slurry in the proportions of each group is relatively small, and the maximum value does not exceed 2%. BC trends in different groups can show that BC is roughly proportional to factor A. BC increases with the increase of factor A, and the reason is similar to the influence of WSR on VSI and FL.

### 3.5. Stone Body Strength (CS and FS)

**Figure 8:**
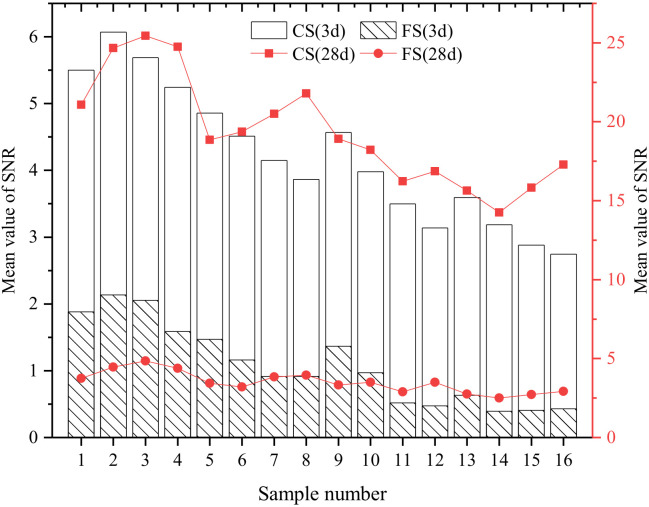
Results of the orthogonal test of stone body strength and the mean value of SNR.

The stone body strength of the grouting material is an important index to evaluate its performance after consolidation, which directly affects the grouting treatment effect of the injected rock and soil. General engineering requirements for grouting materials should have the highest possible strength. For water blocking projects, grouting materials are also required to have good early strength and erosion resistance. The results of the orthogonal test of stone body strength and the mean value of SNR are shown in Figure 8.

The SR analysis of CS was taken as an example to represent the stone body strength, as shown in Table 8. According to the SR analysis results, the order of the influence of the five factors on CS (3 d) is: A > B > D > C > E. The order of the influence of the five factors on CS (28 d) is: A > E > D > B > C. Among them, A was the most significant factor affecting the stone body strength.

**Figure 9:**
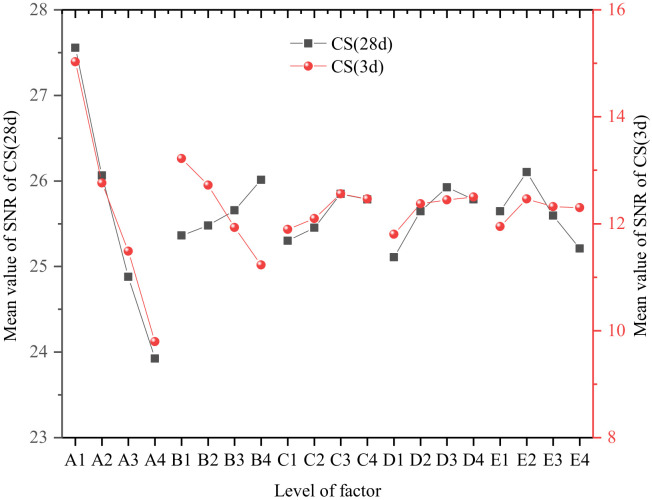
Mean value of the SNR of CS (3 d) and CS (28 d) at different factor levels.

Analysis of Figure 9 shows that Factor A is the most important factor affecting the mechanical properties of hardened cement slurry. The main reason is that the larger the WSR, the more remaining free water there is due to the limited hydration reaction of each component material in the composite slurry. Free water will generate more pores, resulting in a decrease in the compactness of the consolidated body and a decrease in CS. Under the premise of meeting the requirements of grouting engineering, the WSR of the grout should be reasonably selected. With the increase of PS content, CS at different ages showed an increasing trend. When the dosage exceeds the saturated dosage point, the increasing trend of stone body strength slows down. This is mainly due to the surface activity of PS. When the dosage is too large, a large number of air bubbles will be generated in the mixed slurry, which will lead to an increase in the number of harmful pores in the stone body and affect its strength. Therefore, it is necessary to control the content of PS reasonably.

Stone body strength decreased with increasing FIPA. This is because the low activity of FIPA cannot participate in the early hydration reaction, and the internal incorporation of FIPA will reduce the relative proportion of cement in the slurry. Therefore, FIPA acts as an inert component in the early stage, which leads to the reduction of early strength. However, with the progress of the hydration reaction, the volcanic ash activity of FIPA is stimulated, and the strength of the stone body increases in the later stage, which is consistent with the results in the literature [31]. Different from FIPA, the incorporation of SF does not only improve the early strength of the stone body, but also improves the later strength. The reasons are that, on the one hand, the particle size of SF is very small, which can fill the voids of the particles and the pores of the cement slurry, increase the density of the slurry and thus improve its strength. On the other hand, the main chemical composition of SF is amorphous SiO_2_, which is amorphous with high fineness and has strong pozzolanic activity. When the hydration reaction occurs, SF can undergo secondary hydration reaction with the cement hydration product Ca(OH)_2_ to generate C-S-H gel. At the same time, the content of Ca(OH)_2_ in the slurry can be reduced, and the hydration reaction can be forwarded to generate a gel and improve the mechanical properties of cement slurry consolidation. However, when the content of SF is too large, since the specific surface area of SF is much larger than that of cement, the water demand for hydration of the slurry is greater than the molecular weight of water contained in the slurry and the hydration reaction is limited, and the materials that do not participate in the reaction become inert components, so the stone body strength has decreased.

In addition, the mean value of the SNR of CS increased first and then decreased with the increase of CH content. The reason is the filling effect of CH; as a nanoscale material it can be filled in the pores of the particles and the pores of the hydration products, thereby improving the microstructure of the slurry stone body. CH and the surrounding hydration products are bonded to the surface to form C-S-H gel, and with CH as the crystal nucleus, the C-S-H gel is connected into a stronger three-dimensional network structure [32] and the strength of the slurry stone body is improved.

The influence mechanism of the content level of each factor on the grouting material FS is similar to that of CS, so it is not repeated here.

### 3.6. Optimal Analysis of Grouting Material Ratio

The aforementioned studies have analyzed the SNR effect of each factor level on the slurry performance. During the analysis process, it can be found that the optimal slurry ratio of each performance index is not the same. Therefore, it is impossible to obtain a set of ratios that makes each performance index have better results. Therefore, the performance indicators of the grout were comprehensively analyzed using the improved Taguchi method to obtain the best mix ratio of the new cement-based composite grouting material.

Based on the SNR sequence obtained by processing the experimental data using the Taguchi method, the analysis was carried out according to the grey relational analysis steps of the Taguchi method. Table 9 shows the SNR normalized value. Table 10 shows the loss function values. Table 11 shows relevant parameters of the Taguchi method. It can be seen from Table 12 that the maximum grey correlation value can be obtained under the ratio of A3 group, which shows the highest stone body strength, but the flow characteristics and coagulation characteristics are not very good. Therefore, this ratio may not be the optimal ratio for grouting materials. To obtain the optimal ratio for overall performance, the average grey relational degree of all factors was calculated (Table 12). The level with the largest mean grey relational degree is the best of all levels for this factor, as it has the highest main effect on the response [33].

In this paper, the objective weight of the evaluation index is determined by the entropy method of objective weighting, and the weight coefficient (Table 13) analysis is introduced to improve the calculation method of the grey relational degree.

From Table 13, when the WSR is 0.6, the FIPA content is 20%, the SF content is 10%, the PS content is 0.15%, and the CH content is 1%, each factor has the largest average grey correlation degree. To verify the correctness of the analytical method, the slurry properties of the above ratio (A1B3C3D3E2) were tested as shown in Table 13.

For convenience, the ordinary cement slurries, the A3 group slurries of the orthogonal test, and the above-obtained optimal ratio (A1B3C3D3E2) slurries are named as S1, S2, and S3, respectively.

From Table 13, the properties of the slurry under the proportion obtained by the grey relational analysis based on the Taguchi method are all good, which shows that the analysis method is very effective. The optimized S3 slurry has improved flow properties, coagulation properties and stone body strength. Compared with the existing research on optimized cement slurry [5], the flow properties and mechanical properties of S3 are similar to those of S1, and the BC of S3 is smaller and has better coagulation properties.

### 3.7. Slurry Constitutive Equation

**Figure 10:**
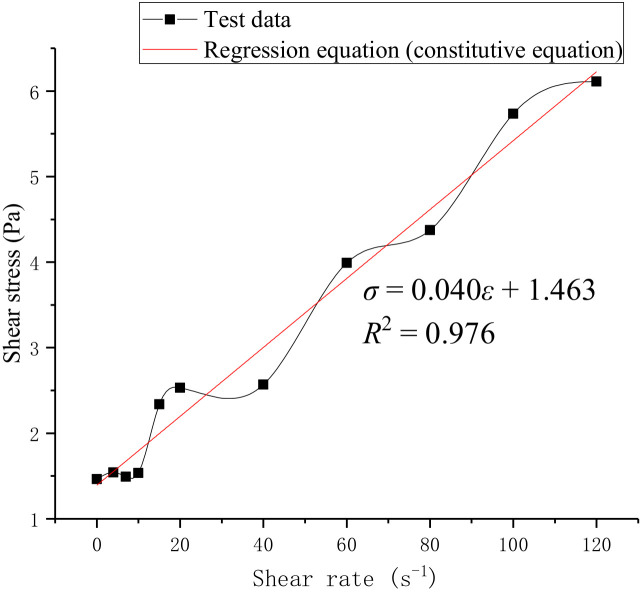
Cement slurry (S3) constitutive model (shear stress-shear rate curve).

According to the VIS test results, the constitutive model (Figure 10) is obtained by further analysis.

*R*^2^ = 0.976, indicating that the regression equation is credible. The intercept of the curve on the Y-axis represents the cohesion of slurry (1.463 Pa), which is also consistent with Wu et al.’s [13] study.

## 4. Discussion

To further study the reasons for the difference in performance between the optimal grout and the ordinary grout, and to support the follow-up study of the cement grout, this section involved microscopic analysis (XRD and SEM) for different grouts, and briefly analyzed the theory of reinforcement mechanical properties.

### 4.1. Analysis of Hydration Hardening Mechanism

#### 4.1.1. Analysis of Hydration Products

**Figure 11:**
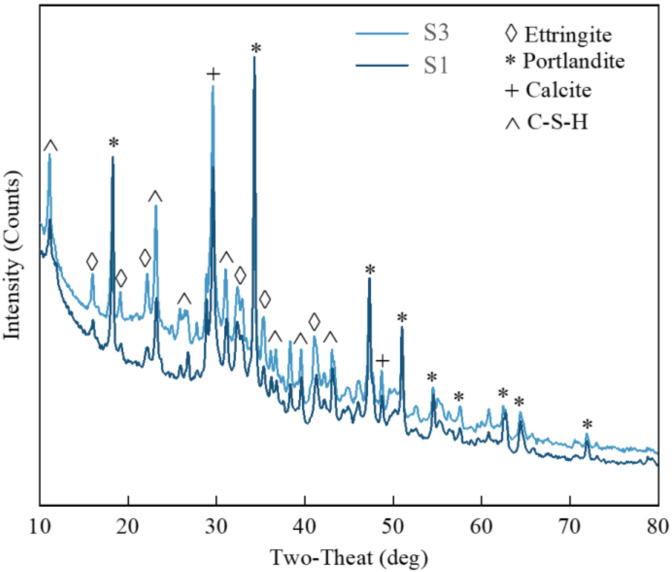
Material composition of grouting material stone body.

The 28 d stone body was taken for XRD testing and the composition of the crystal phase of the stone body was compared and analyzed. Figure 11 shows the XRD patterns of the S3 and the S1.

From Figure 11, the composition of the crystal phase of the two grouting materials is roughly the same, mainly composed of ettringite (Ettringite), Ca(OH)_2_, CaCO_3_ and hydrated silicic acid calcium gel (CSH) composition. Compared with S1, the diffraction intensities of Ettringite and C-S-H peaks of S3 were increased and the content increased. This shows that the hydration degree of the cement-based composite grouting material is higher. Since the secondary hydration reaction between FIPA and silica fume requires the consumption of Ca(OH)_2_, the diffraction intensity of the Ca(OH)_2_ peak of the S3 group is lower than that of the S1 group, and the Ca(OH)_2_ content less.

In addition, it can also be seen that the CaCO_3_ peak diffraction intensity of the S3 group is higher. This is because the CH in the cement-based composite grouting material does not participate in the hydration reaction in the system, but exists in the system by filling and nucleation. There are also CaCO_3_ products generated by the carbonization of calcium hydroxide during the curing and sample preparation process [34], so the content is higher in the phase composition of S3 stone bodies.

#### 4.1.2. Micro-Morphology Analysis

**Figure 12:**
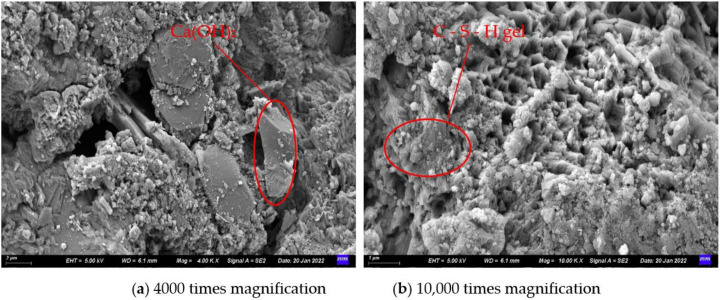
SEM image of grouting material S1.

**Figure 13:**
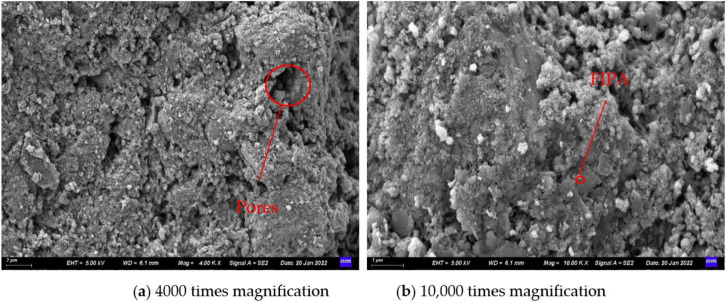
SEM image of grouting material S3.

Adopting SEM technology, the S1 and S3 stone bodies (28 d) were processed and observed. The hydration mechanism of the slurry system was further analyzed by comparing the microscopic morphology of the stone bodies of the S3 and S1 grouting materials. Combined with the previous section, the performance of the stone body was evaluated. Figure 12 and Figure 13 show the microscopic morphologies of the grouting materials S1 and S3.

In Figure 12, many pores and cracks are seen in the SEM image of S1 and the overall structure is relatively loose. In addition, it can also be seen that Ca(OH)_2_ crystals of cement hydration products in the form of hexagonal plates are stacked on each other. Since there are only Portland cement particles in the S1, its hydration products are single, and Ca(OH)_2_ and hydrated calcium silicate gel (C-S-H) are the main hydration phases. The C-S-H gel is the source of the cementitious force of the slurry. Its morphology is greatly affected by clinker activity, solution supersaturation and hydration age, and is generally fibrous, reticulated, granular and flake-like. These hydration products interweave and grow to form a spatial structure, and at the same time leave many pores, resulting in a loose structure of the stone body.

As can be seen from Figure 13, compared with S1, the pores in the stone body structure of S3 become smaller and the plate-shaped hydration product Ca(OH)_2_ crystals are rare. It can be seen that the gel is wrapped in spherical pulverized coal around the ash particles, and the overall structure is denser and firmer. This is due to the addition of supplementary cementitious materials and optimized materials with smaller particle size than ordinary Portland cement in the S3 system. These granular materials have different degrees of influence on the system through their own pozzolanic effect, filling effect, rolling ball effect and crystal nucleation effect. Among them, the supplementary cementing materials FIPA and SF mainly undergo a secondary hydration reaction with the cement hydration product Ca(OH)_2_ to generate calcium silicate hydrate, calcium aluminate hydrate and other products, that is, the pozzolan effect. Therefore, the plate-like hydration product Ca(OH)_2_ crystals are less seen in Figure 13. In addition, due to the small particle size of these materials, the particles that have not undergone hydration reaction can also be filled in the pores and cavities of the hydration product, thereby making the structure of the stone body more compact, which is the filling effect. The form of FIPA is spherical microbeads. Compared with cement, its surface is smoother and the adsorption force of water molecules is small. It can better disperse other particles in the system and accelerate the hydration reaction to generate more hydration and gelling. CH provides an adsorption surface for the nucleation and growth of hydration products, improves the compactness of hydration products, and plays the role of filling and crystallization nucleation in the system.

Comparing S1 and S3, the developed cement-based composite grouting material S3 has more sufficient hydration reaction, more hydration and gelling products are generated, and particles with smaller particle size fill the space formed by the intertwining and overlapping of hydration products. Therefore, the structure of the stone body is denser, and therefore it has better stone body performance.

### 4.2. Theoretical Analysis of Mechanical Properties of Grouting Reinforcement

The reinforcement mechanism of grouting on fractured rock mass is mainly reflected in two aspects: physical action and chemical cementation. After grouting the fractured rock mass, the original voids in the rock mass are filled with the grout. After the grout solidifies, it bears the force between the rock masses, so that the stress state of the rock mass is redistributed to form a stable whole.

Yang and Zhang [35] proposed that the rock reinforcement after grouting is a composite material. According to the “mixing rate” of the elastic modulus of the composite material and the principle of equivalent strain, the constitutive model of the rock reinforcement can be obtained as follows:

(1) $$E=E_{0}\left(I-D\right)+E_{r}D\cdot \eta ,$$

(2) $$\delta =\frac{\sigma_{ef}}{E_{0}}=\frac{\sigma }{E_{0}\left(I-D\right)+E_{r}D\cdot \eta },$$

(3) $$\sigma =\left(I-D+S\right)E_{0}\varepsilon ,$$

Among them, *E* is the elastic modulus of the grouting solid. *E*_0_ is the elastic modulus of the intact rock. *D* is the void ratio matrix. *E_r_* is the elastic modulus of the grouting material stone body. η is the filling coefficient matrix. *S* is the reinforcement factor, which is related to the crack distribution, grouting material and filling degree.

From Equation (3), the elastic modulus of the fractured rock mass after reinforcement is related to the damage degree of the fractured rock mass and the reinforcement factor, which is inversely proportional to the former and proportional to the latter. Between 0 and 1, generally speaking, the grouting material has high consolidation strength, good stability and reasonable grouting process, and the *S* value is larger. The stress distribution in the rock body after grouting reinforcement is uniform, which effectively weakens to avoid stress concentration phenomenon, and the integrity of fractured rock mass can be well improved.

**Figure 14:**
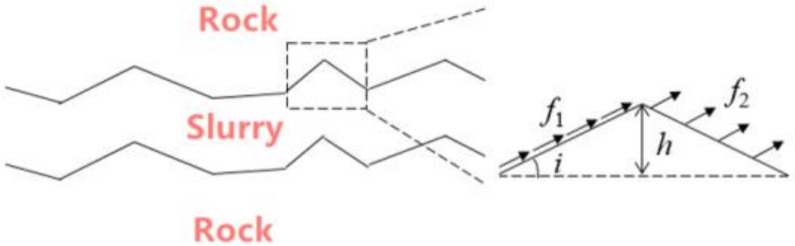
Schematic diagram of grouting and solid shear force.

The failure of grouting-reinforced jointed rock mass mostly occurs along the magma-rock cementation surface, and the jointed rock wall basically remains intact with the failure of local grout cement. To further study the force mechanism of the failure of the grouting-reinforced jointed rock mass, a schematic diagram of the force (Figure 14) was drawn for analysis.

Assuming that the protrusion of the slurry cement in the figure is an isosceles triangle with height *h* and base angle *i*, and the bonding forces of the slurry–rock cement interface are *f*_1_ and *f*_2_, respectively, according to the force balance condition:

(4) $$\left\{\begin{array}{c} \sigma_{n}=\sigma +f_{1}\sin i+f_{2}\sin i\\ \tau_{s}=\tau -f_{1}\cos i-f_{2}\cos i \end{array}\right.,$$

According to the Mohr–Coulomb strength criterion, the shear stress and the normal stress have a linear relationship when the rock mass fails. The set of stress at the failure point is called the Mohr strength envelope, and the shear strength can be expressed as:

(5) $$\tau_{p}=c+\sigma_{n}{\tan\varphi }_{b},$$

After grouting, the cohesion and internal friction angle of the jointed rock mass change. The expression of shear strength after grouting can be transformed into the following formula from Equation (5):

(6) $$\tau_{p}=\left(c+\Delta c\right)+\sigma_{n}{\tan(\varphi }_{b}+\Delta \varphi )=c^{\prime }+\sigma_{n}{\tan\varphi }_{b}^{\prime },$$

Combining Equations (4) and (6), the shear strength of the jointed rock mass after grouting can be obtained as:

(7) $$\tau_{p}=c^{\prime }+f_{1}\cos i+f_{2}\cos+i\left(\sigma +f_{1}\sin i+f_{2}\sin i\right){\tan\varphi }_{b}^{\prime },$$

However, in practice, the bonding force between the front shear surface and the back shear surface of the protrusions on the joint surface does not play the same role, and the superposition analysis cannot be performed simply. The front shear plane is mainly due to the friction force, while the back shear plane is mainly due to the cementation force. From the safety point of view, when calculating the shear strength, only the friction force is considered in the front shear plane, and only the cementation force is considered in the back shear plane. Equation (7) can be further transformed into:

(8) $$\tau_{p}=c^{\prime }+f_{1}\cos i+\left(\sigma +f_{2}\sin i\right){\tan\varphi }_{b}^{\prime },$$

From Equation (8) it is seen that the roughness of the joint surface and the interfacial bonding force can improve the shear strength to varying degrees. From the point of view of grouting, the magnitude of the bond force of the slurry–rock interface is related to the quality of the cementation of the grouting material. Generally, the higher the degree of hydration reaction of grouting materials, the greater the interface bonding force. It is very important to choose grouting materials reasonably in practical engineering.

## 5. Engineering Application Feasibility Verification

To verify the feasibility of the cement slurry in this study under a variety of different engineering conditions, similar materials developed by researchers [20] were adopted with high similarity to the surrounding rock of deep wells, samples of 100 × 100 × 300 mm were fabricated, and fractures were set according to the joint roughness coefficient standard [13] and the grouting equipment [5]. The following three variables were set: (1) Grouting pressure (*σ*_g_, MPa): 2 MPa, 7 MPa and 12 MPa; (2) Confining pressure (*σ*_c_, MPa): 2.5 MPa, 7.5 MPa and 15 MPa; (3) Joint roughness coefficient (*JRC*): 2.5 and 6.5.

### 5.1. Feasibility Study of Flow Characteristics

Through DEM (discrete element method), combined with UDEC software, the obtained parameters of rock-like materials [20] are used to establish rock numerical models of different *JRC*s (the modeling method is similar to the author’s previous research [13], and due to the length of the article it will not be repeated here). Substitute the findings in Section 4.2 into numerical simulations to assign the interface between the cement slurry and the rock. The parameters of S3 in Table 12 and the constitutive equation in Figure 10 were substituted into the numerical simulation software to assign values to the cement slurry, and then the seepage study of the cement slurry was carried out. The volume flow rate is mainly recorded here, denoted as *Q* (10^−6^ m^3^/s).

**Figure 15:**
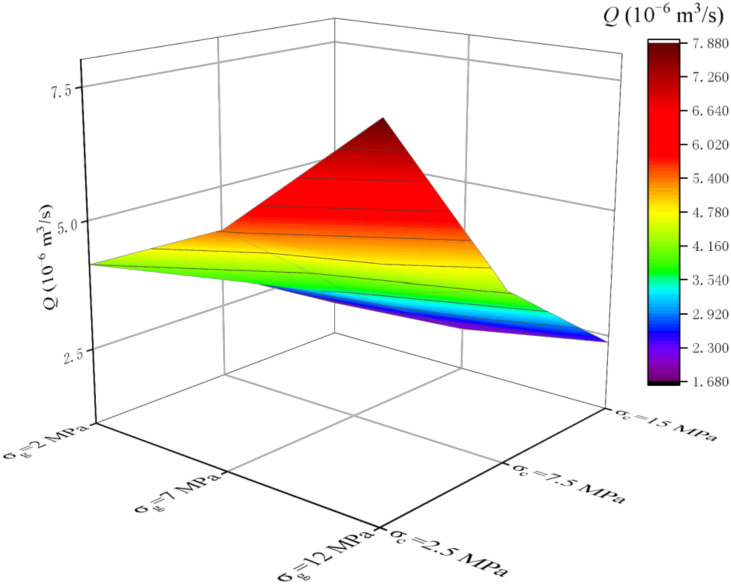
*Q* value of different *σ*_c_ and different *σ*_g_ at *JRC* = 2.5.

**Figure 16:**
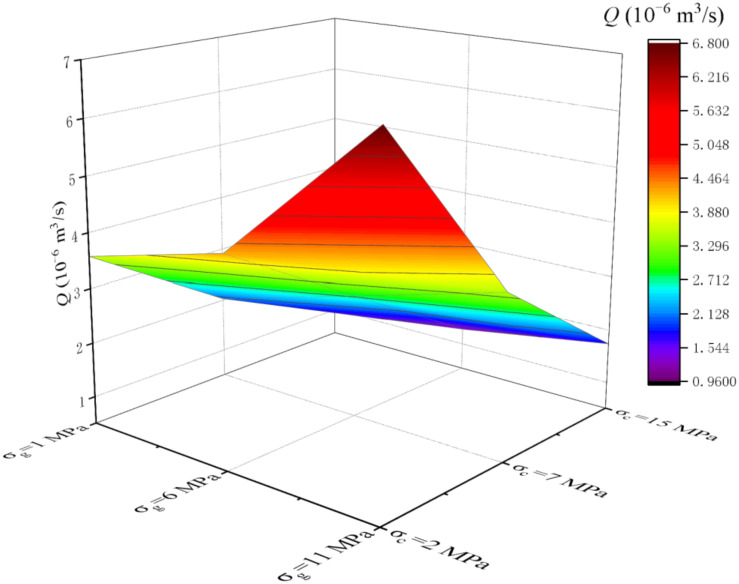
*Q* value of different *σ*_c_ and different *σ*_g_ at *JRC* = 6.5.

The analysis of Figure 15 and Figure 16 shows that *Q* increases gradually with the increase of *σ*_g_, but the increase rate of *Q* slows down with the increase of *σ*_c_. This is mainly because the increase of confining pressure makes the seepage channel narrow, which makes the cement grout less. There is a negative correlation between *Q* and *σ*_c_, but the decrease rate of *Q* slows down with the increase of *σ*_g_. This is mainly due to the increase of *σ*_g_, the seepage channel has a certain trend of opening, so that the cement grout seeping through increased. By comparing Figure 15 and Figure 16, it can be found that with the increase of *JRCs*, *Q* value gradually decreases. This indicates that the more complex the roughness of the fracture is, the more influence it has on the seepage of slurry. This is consistent with previous studies [13], indicating that the cement grout obtained in this study has good flow characteristics.

### 5.2. Different Geological Temperatures

**Figure 17:**
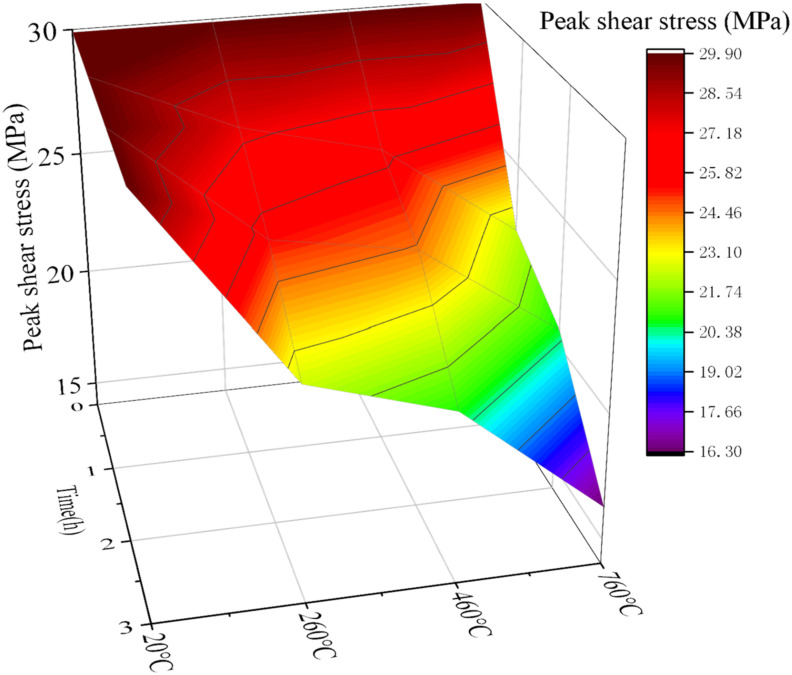
Shear strength of samples under different geological temperature conditions before grouting.

**Figure 18:**
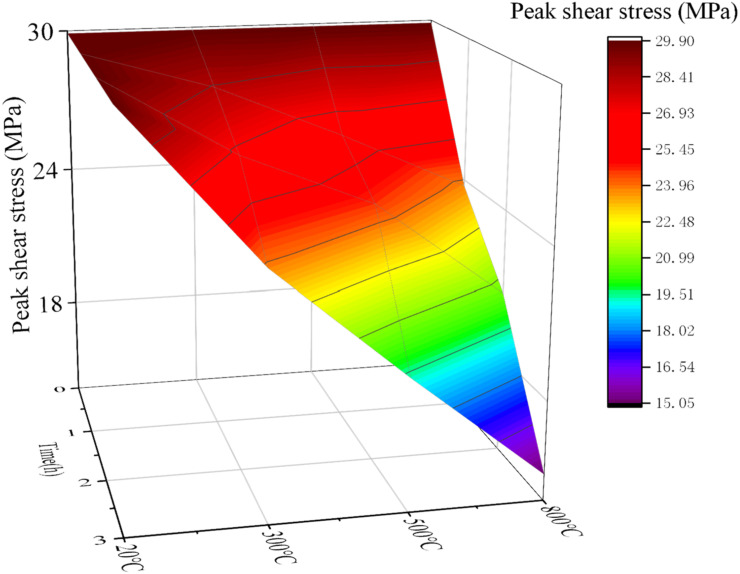
Shear strength of samples under different geological temperature conditions after grouting.

The samples after grouting experiments were subjected to standard curing. Then the shear strength experiments at different temperatures were carried out. The temperature variables were: 20 °C, 260 °C, 460 °C and 760 °C. The time variables were 0 h, 1 h, 2 h, 3 h. Fixed *σ*_c_ = 7.5 MPa, the *σ*_g_ = 7 MPa, and *JRC* = 2.5. Due to many variables, the fixed normal pressure in the shear experiment was *σ_n_* = 6 MPa. The experimental results are shown in Figure 17 and Figure 18.

From Figure 17 and Figure 18, it is seen that with the increase of temperature, the shear strength gradually decreases, and the rate of decrease is larger when the temperature is higher. With the increase of heating time, the shear strength also showed a negative correlation trend, and the decline rate also showed a positive correlation trend with time. Compared with Figure 17 and Figure 18, the shear strength after grouting is slightly improved. However, when the temperature is higher and the heating time is longer, the shear strength decreases rapidly and the minimum value is lower than that before grouting. This is because the cement slurry stone body exhibits poor durability when the temperature is too high. The change trend of this conclusion is consistent with the previous research of other scholars [20]. The accuracy and feasibility of this study have been demonstrated.

### 5.3. Erosion of Different Geological Corrosive Liquids

**Figure 19:**
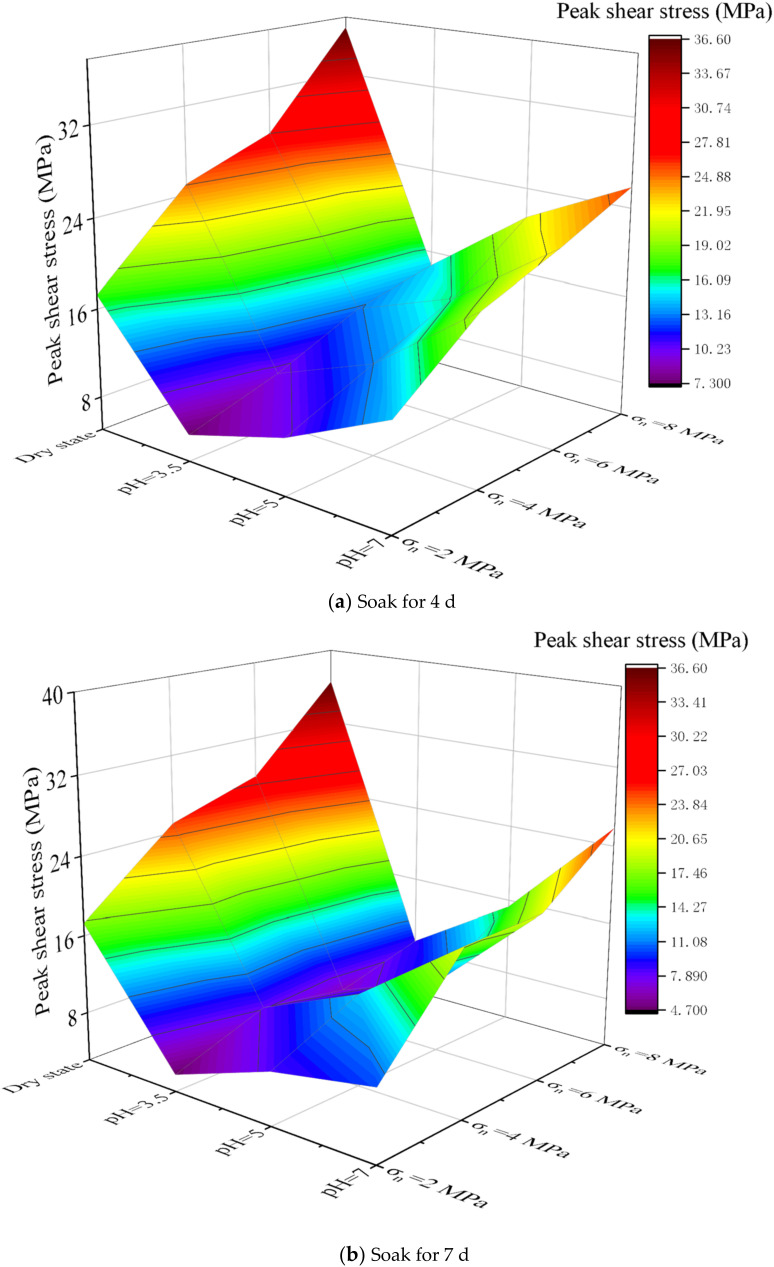
Shear strength of samples at different pH (before grouting).

**Figure 20:**
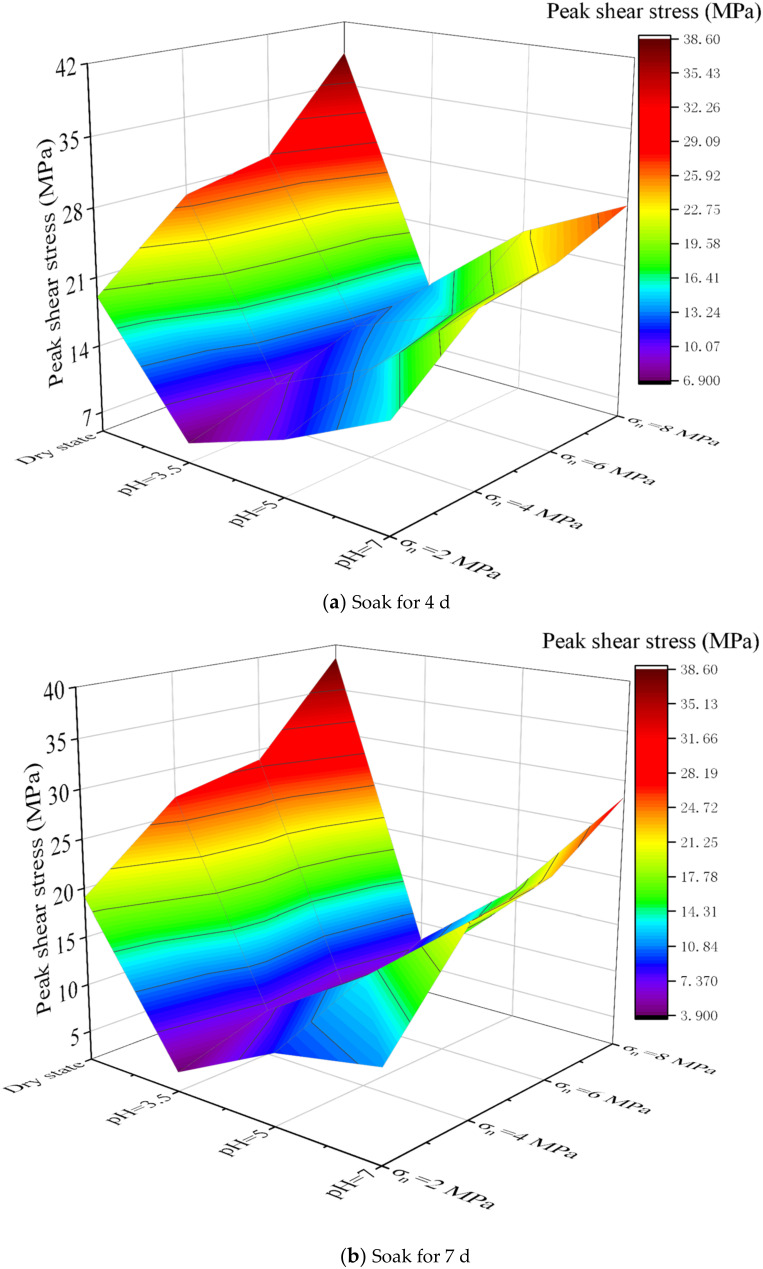
Shear strength of samples at different pH (after grouting).

Different acid-base groundwater erosion studies were carried out on the samples before and after grouting. The solutions with pH of 3.5, 5 and 7 were selected for experiment. The samples were soaked in different solutions for 4 d and 7 d, respectively, and then the shear strength test was carried out. Fixed *σ*_c_ = 7.5 MPa, the *σ*_g_ = 7 MPa, and *JRC* = 2.5. In the shearing experiment, the *σ_n_* were set to 2 MPa, 4 MPa, 6 MPa and 8 MPa, respectively. The experimental results are shown in Figure 19 and Figure 20.

Comparing Figure 19 and Figure 20, the shear strength of the samples decreases with decreasing pH. This indicates that the sample has poor mechanical properties in strong acid solution. However, when the pH is at a lower value, the decrease rate of the shear strength of the sample increases. And the shear strength of the samples after grouting has a slightly higher rate of decrease, which is mainly because the consolidated body of the cement slurry lacks aggregates, and its durability is worse than that of rock-like materials. This is also consistent with the previous study [36] that aggregate has a great influence on rock strength. Different normal pressures can indicate that under different pH conditions, the shear strength of the sample is positively correlated with the normal pressure. This is also in agreement with the conclusions of existing studies [37,38]. Therefore, it also shows that the cement slurry in this study has good reliability and feasibility.

## 6. Conclusions

In this study, the cement slurry was developed using silica fume, which effectively alleviated the environmental problems, and the new slurry was low in cost and superior in performance. The main research conclusions are as follows:

1. The comprehensive application of the entropy weight method and the Taguchi grey relational orthogonal analysis method obtains the optimum ratio of slurry (S3): WSR (0.6), FIPA (20%), SF (10%), PS (0.15%), CH (1%). Compared with pure cement grouting materials, the flow properties, coagulation properties and stone body strength of cement-based composite grouting materials are improved.
2. Compared with the stone body of S1, the overall structure of S3 is more compact and firmer, and the macroscopic mechanical properties are better.
3. The significant relationship of each factor affecting the overall performance of the grouting material is: WSR > PS > FIPA > SF > CH.
4. The developed cement grout can be widely used for grouting reinforcement of rock mass in fissures in various types of engineering construction such as tunnel, subway and mine roadway.

## Figures and Tables

**Table 1:** Chemical composition of each material (%).

Materials	CaO	SiO_2_	Al_2_O_3_	Fe_2_O_3_	SO_3_	MgO	Na_2_O	K_2_O	TiO_2_	LOI
FIPC	61.24	22.51	3.98	4.07	1.21	2.06	0.64	1.15	-	-
FIPA	6.2	38.16	16.19	27.81	2.04	1.16	1.08	1.51	0.78	1.34
SF	0.37	93.12	1.01	1.02	-	0.68	-	-	-	-

**Table 2:** Basic properties of CH.

Exterior	Odor	Proportion	pH	Average Particle Size	Quality Score
White powder	Odorless	2.6	9.2	28 nm	≥99.9%

**Table 3:** Experimental factor level.

Level	Factor				
	A	B (%)	C (%)	D (%)	E (%)
1	0.6	10	6	0.05	0.5
2	0.7	15	8	0.1	1
3	0.8	20	10	0.15	1.5
4	0.9	25	12	0.2	2

**Table 4:** Experimental factor level table L_16_(4^5^).

Number	Scheme Combination	A	B	C	D	E
#1	A_1_B_1_C_1_D_1_E_1_	1	1	1	1	1
#2	A_1_B_2_C_2_D_2_E_2_	1	2	2	2	2
#3	A_1_B_3_C_3_D_3_E_3_	1	3	3	3	3
#4	A_1_B_4_C_4_D_4_E_4_	1	4	4	4	4
#5	A_2_B_1_C_2_D_3_E_4_	2	1	2	3	4
#6	A_2_B_2_C_1_D_4_E_3_	2	2	1	4	3
#7	A_2_B_3_C_4_D_1_E_2_	2	3	4	1	2
#8	A_2_B_4_C_3_D_2_E_1_	2	4	3	2	1
#9	A_3_B_1_C_3_D_4_E_2_	3	1	3	4	2
#10	A_3_B_2_C_4_D_3_E_1_	3	2	4	3	1
#11	A_3_B_3_C_1_D_2_E_4_	3	3	1	2	4
#12	A_3_B_4_C_2_D_1_E_3_	3	4	2	1	3
#13	A_4_B_1_C_4_D_2_E_3_	4	1	4	2	3
#14	A_4_B_2_C_3_D_1_E_4_	4	2	3	1	4
#15	A_4_B_3_C_2_D_4_E_1_	4	3	2	4	1
#16	A_4_B_4_C_1_D_3_E_2_	4	4	1	3	2

**Table 5:** Experimental factor level table L_16_(4^5^).

Sample No		FL/cm	ST/min	VSI/mPa·s	BC/%	CS/MPa		FS/MPa	
						3 d	28 d	3 d	28 d
A	1	15.35	415	1790.7	0.20	5.501	21.083	1.882	3.739
	2	16.9	450	1312.2	0.05	6.072	24.673	2.137	4.456
	3	18	435	1160.8	0.10	5.688	25.450	2.055	4.857
	4	21.55	440	1028.5	0.50	5.243	24.750	1.588	4.389
B	1	22.95	435	725.4	0.75	4.857	18.857	1.471	3.441
	2	30.65	460	162.2	1.30	4.511	19.374	1.162	3.203
	3	16.4	455	1432.5	0.10	4.148	20.503	0.915	3.835
	4	23.15	470	646.5	0.90	3.867	21.797	0.914	3.944
C	1	24.9	500	487.3	0.70	4.567	18.922	1.367	3.329
	2	24.85	490	493.6	0.80	3.980	18.213	0.970	3.496
	3	30.9	505	110.4	1.25	3.497	16.231	0.517	2.884
	4	21.7	510	968.8	0.80	3.138	16.869	0.475	3.495
D	1	25.3	490	397.2	0.70	3.594	15.642	0.633	2.746
	2	22.8	510	790.6	0.60	3.186	14.259	0.392	2.497
	3	32.5	530	45.4	1.80	2.881	15.826	0.410	2.724
	4	31.85	565	59.9	1.50	2.744	17.289	0.432	2.929

**Table 6:** SR analysis of the SNR of FL and VSI.

Factor		A	B	C	D	E
FL	K1	25.0136	26.7311	28.3280	25.4767	27.2893
	K2	27.1330	27.3379	27.1851	27.4276	26.7102
	K3	28.0897	27.3598	26.8698	27.5725	27.4064
	K4	28.8802	27.6878	26.7337	28.6398	27.7106
	SR	3.867	0.957	1.594	3.163	1.000
VSI	K1	62.2399	57.0020	46.4173	61.4665	52.0701
	K2	55.1865	54.5969	53.1093	52.8527	53.6966
	K3	52.0519	49.6044	57.3054	51.9807	54.3003
	K4	44.6572	52.9322	57.3034	47.8355	54.0685
	SR	17.583	7.398	10.888	13.631	2.230

**Table 7:** SR analysis of the SNR of ST.

Factor		A	B	C	D	E
ST	K1	52.7660	53.2285	53.6807	53.4560	53.5231
	K2	53.1568	53.5687	53.6177	53.5940	53.8112
	K3	54.0001	53.6203	53.5856	53.5961	53.4951
	K4	54.3705	53.8758	53.4093	53.6473	53.4640
	SR	1.604	0.647	0.271	0.191	0.347

**Table 8:** SR analysis of the SNR of CS.

Factor		A	B	C	D	E
3 d	K1	14.9915	13.2101	11.8838	11.7904	11.9364
	K2	12.7287	12.7035	12.1293	12.3496	12.4955
	K3	11.4992	11.8800	12.5257	12.3977	12.3064
	K4	9.7836	11.2093	12.4641	12.4652	12.2647
	SR	5.208	2.001	0.642	0.675	0.559
28 d	K1	27.5771	25.3534	25.2963	25.0847	25.6103
	K2	26.0644	25.4696	25.4707	25.6761	26.0938
	K3	24.8738	25.6359	25.8757	25.8966	25.5714
	K4	23.9276	25.9841	25.8003	25.7856	25.1674
	SR	3.649	0.631	0.579	0.812	0.926

**Table 9:** SNR normalized value.

No.	VSI	FL	ST	BC	CS (3 d)	CS (28 d)	FS (3 d)	FS (28 d)
A1	1.00	0.00	0.00	0.90	0.88	0.68	0.93	0.61
A2	0.92	0.13	0.23	1.29	1.00	0.95	1.00	0.87
A3	0.88	0.21	0.34	1.09	0.92	1.00	0.98	1.00
A4	0.85	0.45	0.23	0.64	0.82	0.95	0.82	0.85
B1	0.75	0.54	0.19	0.53	0.72	0.48	0.78	0.48
B2	0.35	0.92	0.37	0.38	0.63	0.53	0.64	0.37
B3	0.94	0.09	0.37	1.09	0.52	0.63	0.50	0.64
B4	0.72	0.55	0.44	0.48	0.43	0.73	0.50	0.69
C1	0.65	0.64	0.64	0.55	0.64	0.49	0.74	0.43
C2	0.65	0.64	0.71	0.51	0.47	0.42	0.53	0.51
C3	0.24	0.93	0.51	0.39	0.31	0.22	0.16	0.22
C4	0.83	0.46	0.61	0.51	0.17	0.29	0.11	0.51
D1	0.59	0.67	0.71	0.55	0.34	0.16	0.28	0.14
D2	0.78	0.53	0.58	0.59	0.19	0.00	0.00	0.00
D3	0.00	1.00	0.83	0.29	0.06	0.18	0.03	0.13
D4	0.08	0.97	0.92	0.34	0.00	0.33	0.06	0.24

**Table 10:** Loss function values.

No.	VSI	FL	ST	BC	CS (3 d)	CS (28 d)	FS (3 d)	FS (28 d)
A1	0.00	1.00	1.00	1.90	0.12	0.32	0.07	0.39
A2	0.08	0.87	0.77	2.29	0.00	0.05	0.00	0.13
A3	0.12	0.79	0.66	2.09	0.08	0.00	0.02	0.00
A4	0.15	0.55	0.77	1.64	0.18	0.05	0.18	0.15
B1	0.25	0.46	0.81	1.53	0.28	0.52	0.22	0.52
B2	0.65	0.08	0.63	1.38	0.37	0.47	0.36	0.63
B3	0.06	0.91	0.63	2.09	0.48	0.37	0.50	0.36
B4	0.28	0.45	0.56	1.48	0.57	0.27	0.50	0.31
C1	0.35	0.36	0.36	1.55	0.36	0.51	0.26	0.57
C2	0.35	0.36	0.29	1.51	0.53	0.58	0.47	0.49
C3	0.76	0.07	0.49	1.39	0.69	0.78	0.84	0.78
C4	0.17	0.54	0.39	1.51	0.83	0.71	0.89	0.49
D1	0.41	0.33	0.29	1.55	0.66	0.84	0.72	0.86
D2	0.22	0.47	0.42	1.59	0.81	1.00	1.00	1.00
D3	1.00	0.00	0.17	1.29	0.94	0.82	0.97	0.87
D4	0.92	0.03	0.08	1.34	1.00	0.67	0.94	0.76

**Table 11:** Grey relational coefficients and grey relational grade values.

No.	Grey Relational Coefficients								Grey Relational Grade	Grey Relational Order
	VSI	FL	ST	BC	CS (3 d)	CS (28 d)	FS (3 d)	FS (28 d)		
A1	1.0000	0.3333	0.333	0.2085	0.8010	0.6061	0.8698	0.5598	0.576	4
A2	0.8553	0.3645	0.393	0.1795	1.0000	0.9032	1.0000	0.7941	0.692	2
A3	0.8091	0.3883	0.430	0.1929	0.8586	1.0000	0.9562	1.0000	0.716	1
A4	0.7682	0.4772	0.393	0.2334	0.7302	0.9122	0.7407	0.7665	0.635	3
B1	0.6703	0.5188	0.382	0.2464	0.6402	0.4914	0.6944	0.4912	0.516	8
B2	0.4335	0.8649	0.444	0.2665	0.5720	0.5150	0.5822	0.4442	0.528	6
B3	0.8917	0.3542	0.444	0.1929	0.5103	0.5726	0.5000	0.5846	0.501	11
B4	0.6433	0.5251	0.473	0.2527	0.4681	0.6515	0.4998	0.6149	0.522	7
C1	0.5854	0.5847	0.584	0.2441	0.5824	0.4943	0.6552	0.4682	0.531	5
C2	0.5878	0.5829	0.631	0.2486	0.4846	0.4640	0.5180	0.5029	0.510	10
C3	0.3974	0.8814	0.505	0.2650	0.4186	0.3917	0.3742	0.3896	0.466	13
C4	0.7494	0.4815	0.562	0.2486	0.3756	0.4133	0.3607	0.5028	0.459	14
D1	0.5496	0.5996	0.631	0.2441	0.4309	0.3731	0.4107	0.3685	0.457	15
D2	0.6921	0.5141	0.542	0.2390	0.3811	0.3333	0.3333	0.3333	0.418	16
D3	0.3333	1.0000	0.749	0.2801	0.3476	0.3788	0.3393	0.3652	0.494	12
D4	0.3510	0.9489	0.866	0.2723	0.3333	0.4283	0.3466	0.3967	0.515	9
Weight coefficient	0.2	0.2	0.1	0.35	0.15	0.15	0.15	0.15	-	-

**Table 12:** Average grey relational grade for each level of different factors. Note: The optimum level of each experimental parameter is marked with (*).

Level	A	B	C	D	E
1	0.655 *	0.520	0.522	0.488	0.526
2	0.517	0.537	0.540	0.534	0.560 *
3	0.492	0.544 *	0.547 *	0.564 *	0.540
4	0.471	0.533	0.525	0.547	0.509

**Table 13:** Comparison of slurry performance under different conditions.

No.	FL/cm	VSI/mPa·s	ST/min	BC/%	CS/MPa		FS/MPa	
					3 d	28 d	3 d	28 d
S1	22.6	657.1	570	3	5.741	23.533	3.368	5.560
S2	18	1160.8	435	0.1	5.688	25.450	2.055	4.857
S3	25.4	459.6	415	1.5	5.965	26.144	2.703	5.095

## Data Availability

All data, models and code generated or used during the study appear in the submitted article.
